# Purification of quantum dot-based bioprobes with a salting out strategy†

**DOI:** 10.1039/d1na00569c

**Published:** 2022-01-04

**Authors:** Zhi-Liang Chen, Jia-quan Xu

**Affiliations:** School of Pharmacy, Shaoyang University Shaoyang 422000 P. R. China; Jiangxi Key Laboratory for Mass Spectrometry and Instrumentation, East China University of Technology Nanchang 330013 China jiaquan_xu@foxmail.com +86-739-5308282

## Abstract

A salting out strategy is reported for purification of IgG-conjugated QD (IgG-QD) bioprobes. Adding NaCl can precipitate free IgG selectively, while the IgG-QD maintains good colloidal stability. The dynamic light scattering technique reveals that this is due to the relatively positive zeta potential of free IgG than that of the IgG-QD.

## Abstract 2

A salting out strategy is reported for purification of IgG-conjugated QD (IgG-QD) bioprobes. The optical properties, target recognition, and colloidal stability of the purified IgG-QD were commendably maintained after salting out.

## Graphical abstract

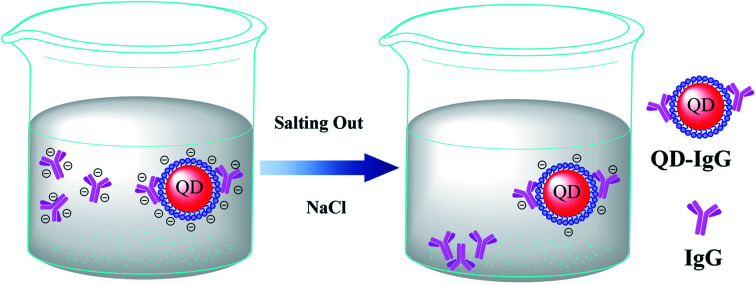

Quantum dots (QDs) with outstanding optical properties have been employed to prepare bioprobes for biolabelling, bioimaging and biosensing.^1–3^ Generally, both QDs synthesized in the aqueous phase or the oil phase have no target recognition function. A prerequisite for the possible bioapplication of QDs is the proper surface functionalization through incorporating target biological moieties, such as an antibody, polypeptide, deoxyribonucleic acid (DNA), *etc.*^4–6^ Unfortunately, in the process of functionalization, a large number of biomolecules in the free state co-exist in the biomolecule-conjugated QD probe solution, which is undesirable for the further use of QDs. To address this problem, size exclusion chromatography (SEC) is usually employed to separate the free biomolecules in biomolecule-conjugated QD bioprobe solution.^7,8^ Nonetheless, the SEC-based purification strategy is still dissatisfactory due to its complicated procedures, time-consuming nature, and low purification throughput. Importantly, the high pressure of SEC may affect the bio-activity of biomolecules according to previous reports.^9–11^ For example, as early as the beginning of the 20th century, Bridgman's results showed that a pressure of 7 kbar can denature the proteins of egg white similarly to temperature.^10^ Gabellieri's results demonstrated that dynamic high pressure causes changes in the supramolecular structure of soy proteins.^11^ Therefore, it is of great significance to develop a strategy under the mild conditions and facile process for the purification of QD-based bioprobes.

Salting out describes the precipitation of less soluble samples from a mixture solution after adding electrolytes such as sodium chloride (NaCl), potassium chloride (KCl), magnesium chloride (MgCl_2_), *etc.*^12^ After two centuries of progress, scholars have employed salting out to separate chemical and biological samples such as proteins, polypeptides, DNA, *etc.*^13–15^ Recently, Schroit *et al.* reported a salting out strategy to isolate tumor-derived exosomes with acetate by using charge neutralization.^16^ Lee *et al.* developed a salting out strategy to sequentially separate graphene oxides by varying the ammonium sulfate concentrations.^17^ Foster *et al.* reported that the yeast enzymes can be precipitated by using ammonium sulfate.^18^ Gan *et al.* presented a salting out a strategy to isolate DNA from whole blood.^19^ Ryall *et al.* reported a salting out strategy to precipitate the calcium oxalate in undiluted human urine using urate.^20^ In our previous work, salting out with NaCl has been applied to separate the octylamine-*grafted* poly-(acrylic acid) (OPA) micelles in OPA-coated QDs solution.^21^

Herein, inspired by the above-mentioned studies, salting out is used to separate free Immunoglobulin G (IgG) in IgG-conjugated QD (IgG-QD) solution. As illustrated in Fig. 1a, the addition of NaCl can preferentially compress the zeta potential of free IgG to electrical neutrality due to the relatively positive zeta potential of free IgG, resulting in the aggregation and precipitation of free IgG, while the IgG-QD still maintains good colloidal stability due to its relatively negative zeta potential. Therefore, the separation of free IgG in IgG-QD solution can be achieved effectively by adding an appropriate concentration of NaCl.

**Fig. 1:**
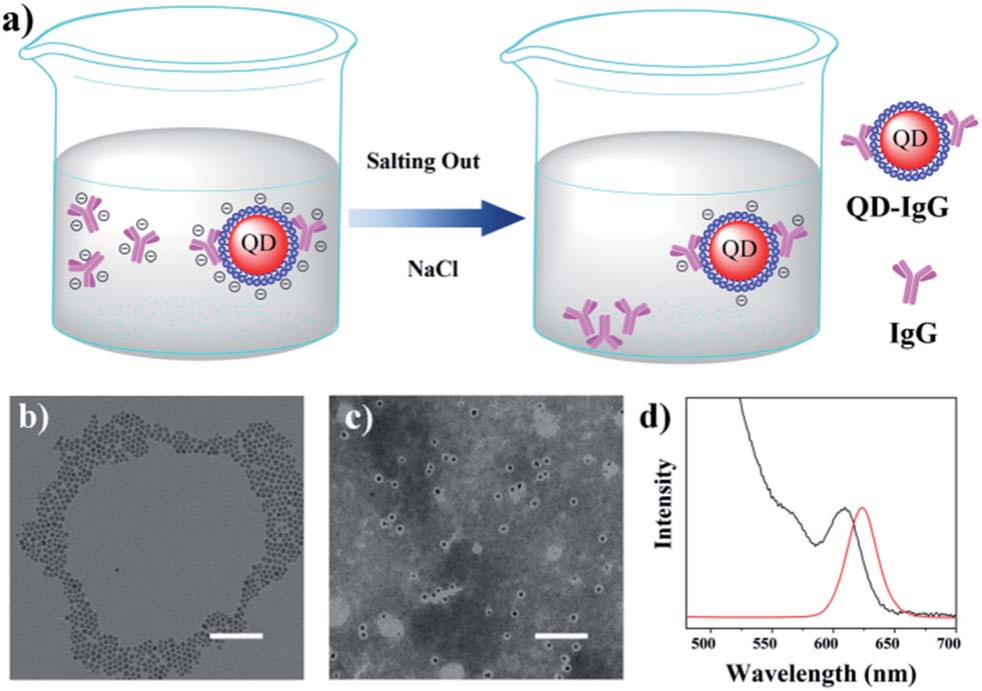
(a) Purification strategy of IgG-QD solution with salting out, and TEM images of (b) hydrophobic and (c) hydrophilic QDs, and the scale bar is 100 nm. (d) Absorption (black) and PL emission (red) spectra of hydrophilic QDs.

The hydrophobic CdSe/CdS QDs were prepared by following our previous method.^8,21^ As shown in Fig. 1b, the transmission electron microscopy (TEM) image of the as-prepared QDs illustrated their uniform diameter of 7.3 ± 1.2 nm (Fig. S1†). Subsequently, OPA was synthesized (Fig. S2†) and utilized to prepare hydrophilic OPA-coated QDs (OPA-QDs). As shown in Fig. S3,† the prepared hexane-dispersed QDs were transferred to water solution after adding OPA, indicating that the hydrophilic OPA-QDs were prepared successfully. After that, the OPA-QDs were purified with NaCl to remove the OPA micelles according to our previously reported strategy.^21^ The TEM picture of the purified OPA-QDs displayed in Fig. 1c shows that with the assistance of phosphotungstic acid (1%), no empty OPA micelles can be observed in the NaCl-treated OPA-QDs, suggesting that the OPA micelles were separated from the OPA-QD solution successfully. The photoluminescence (PL) spectrum showed that the as-prepared OPA-QDs emit red fluorescence at 628 nm (Fig. 1d). Then, amine-PEG-carboxyl (NH_2_-PEG-COOH, MW = 2000) reacted with the carboxyl of OPA-QDs to obtain OPA-QDs-PEG for the further bioconjugation and the decrease of non-specific adsorption. As shown in Fig. S4,† after reacting with NH_2_-PEG-COOH, the electrophoretic speed of OPA-QDs-PEG is significantly slower than that of OPA-QDs, indicating that NH_2_-PEG-COOH was grafted on OPA-QDs successfully. Next, the resulting OPA-QDs-PEG (3 μmol, 200 μL) was conjugated to 1 mg of IgG (anti-mouse second antibody, 1 mL) to obtain the IgG-QD probes.

Subsequently, we added 1 mL of NaCl (2 mol L^−1^) into IgG-QD (3 μmol L^−1^, 200 μL) solution, and the IgG-QD solution (Fig. S5,† left) became turbid rapidly (Fig. S5,† middle) and also could be changed to transparent again after 2 h (Fig. S5,† right). This is in agreement with our previous results that the turbid NaCl-treated OPA-QD solution can be recovered to transparent again, indicating that the free IgG can be precipitated selectively from IgG-QD solution after adding NaCl. The purification results were characterized by SEC. As can be seen in Fig. 2a–c, the retention time of free IgG (Fig. 2a) and IgG-QD (Fig. 2b) is 33 min and 24 min, respectively. After salting out with NaCl, the chromatographic peak of the free IgG is disappeared for the IgG-QD solution (Fig. 2c), suggesting that the free IgG was removed from the IgG-QD solution. It should be noted that a high IgG to IgG-QD ratio and the electrolyte with an appropriate type and concentration are beneficial for the selective precipitation of free IgG from IgG-QD solution.

**Fig. 2:**
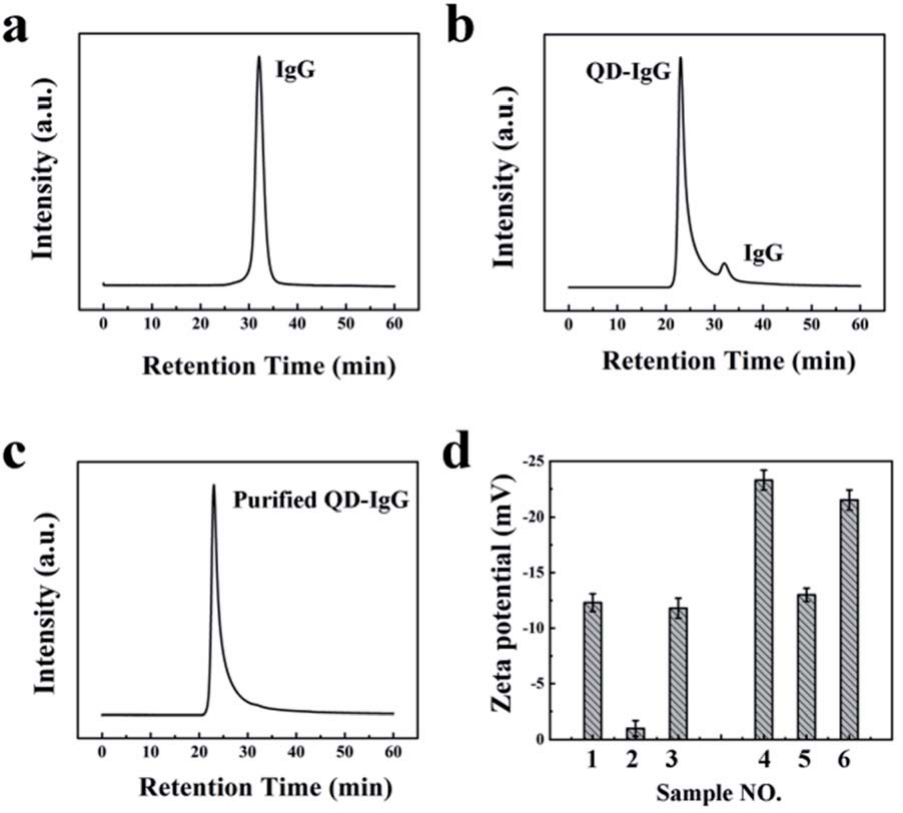
(a–c) Chromatogram of IgG (a), and IgG-QD (b) before and (c) after adding NaCl. (d) Zeta potential of the IgG-QD and IgG before and after salting out (1–3: IgG, 1: no NaCl; 2: adding NaCl; 3: removing NaCl; 4–6: IgG-QD: 4: no NaCl; 5: adding NaCl; 6: removing NaCl).

The purified IgG-QD was collected and dispersed in ultrapure water by using a centrifugal filter device to remove NaCl for the dynamic light scattering (DLS) measurements. The zeta potential of IgG and IgG-QD probes was investigated by DLS before and after adding NaCl. As shown in Fig. 2d, after the addition of NaCl (2 mol L^−1^), the zeta potential of free IgG increased from −12 mV (Fig. 2d, sample 1) to *ca.* 0 (Fig. 2d, sample 2) and can be recovered to −13 mV (Fig. 2d, sample 3) after removing NaCl with the centrifugal filter device.^22^ Similarly, IgG-QD probes increased from −32 mV (Fig. 2d, sample 4) to −16 mV (Fig. 2d, sample 5), and can also return to −30 mV (Fig. 2d, sample 6) after NaCl is removed.^23^ The results of Fig. 2d suggested that the free IgG can be preferentially compressed to electrical neutrality due to the zeta potential of free IgG being closer to zero than that of IgG-QD probes after the addition of electrolytes, and the hydration layer of the free IgG can also be destroyed which can induce the aggregation and selective precipitation of free IgG from IgG-QD solution. However, IgG-QD probes still maintain good colloidal stability under the identical salting out conditions due to their relatively negative zeta potential. Subsequently, gel electrophoresis was utilized to characterize the IgG-QD probes before and after NaCl treatment. As shown in Fig. S6,† the electrophoretic speed of NaCl-treated IgG-QD solution is slower than that of IgG-QD probes noticeably, indicating that the surface charge of IgG-QD probes can be increased nearly to zero with the addition of NaCl, which is in agreement with our previous results.^21^ Based on the above results and our previous reports, we assert that the zeta potential of both the free IgG and IgG-QD probes is compressed simultaneously, while IgG with a relatively positive zeta potential is preferentially compressed to electrical neutrality and is precipitated, thus the separation of free IgG in IgG-QD solution is achieved through adding NaCl. Based on the previously reported studies,^24^ we speculate that other electrolytes (*e.g.* KCl and (NH_4_)_2_SO_4_) should also be used for the purification.

The optical properties of IgG-QD solution were also examined before and after treating with NaCl solution. As shown in Fig. S7,† both the absorption and PL spectra of purified QDs, OPA-QDs and IgG-QD exhibited identical profiles before and after being treated with NaCl, indicating that the integrity of QDs was maintained in the process of salting out. Moreover, adding NaCl produced a negligible influence on the quantum yield (QY) (Fig. S8†) compared with that of OPA-QD solution, suggesting that the surface structure of QDs was maintained compared with that of original samples.

Next, the IgG-QD with green PL emission was prepared and utilized to recognize the cytokeratin 8/18 (CK8/18) antigens which are a member of the cytokeratin family, and their expressions are associated with breast cancer and are utilized to monitor the treatment process in breast cancer.^25^ The red-emission IgG-QD was then utilized to recognize the P63 antigens which are a member of the p53 gene family, and their germline mutations are associated with human mammary cancer.^26^ As illustrated in Fig. 3a and c, green fluorescence was localized in the cytomembrane, which is consistent with the previous reports, suggesting that the CK8/18 antigens are expressed in the cytomembrane.^27^ As illustrated in Fig. 3b and d, red fluorescence was localized in the cell nucleus, which is consistent with the previous results,^28^ suggesting that the salting out process exhibits a negligible influence on the target recognition of QD-probes.

**Fig. 3:**
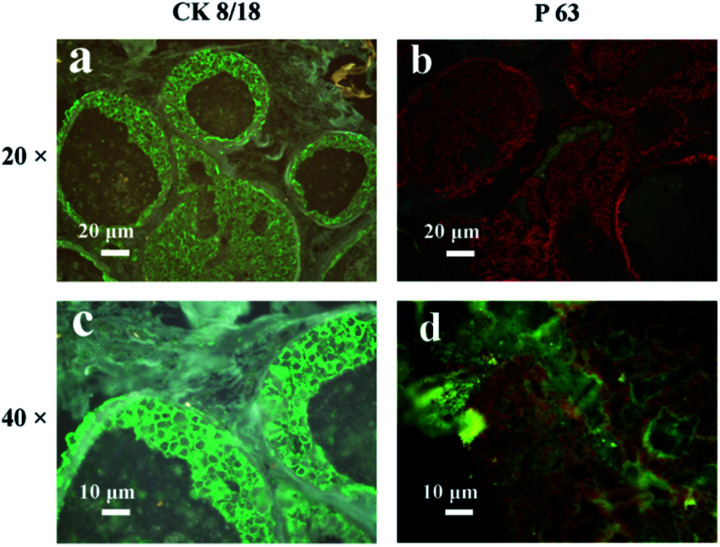
Labeling of CK8/18 (a and c) and P63 (b and d) antigens with purified IgG-QD probes in breast cancer tissue.

Compared with the conventional purification strategy of IgG-QD bioprobes, three main advantages of our salting out strategy are to be highlighted. First, the purification of IgG-QD bioprobes can be achieved only by adding an appropriate amount of NaCl solution, thus the purified process is time- and labor-saving. Second, no supplementary instruments, such as the high performance liquid chromatograph and ultracentrifuge, are required, thus the purification can be realized in a general laboratory. Last and the most important, the biological function of QD-based probes can be maintained after purification because of the mild purification conditions without high pressure and high speed centrifugation, which is beneficial for maintaining the target recognition of the IgG-QD.

In conclusion, a purification strategy of IgG-QD bioprobe solution containing free IgG is presented based on a “charge neutralization” strategy. After treating NaCl, the free IgG can be aggregated and precipitated from IgG-QD solution. Using zeta potential analytical techniques, we have revealed that by adding NaCl, the relatively positive zeta potential of free IgG is compressed to electrical neutrality preferentially, which can induce the aggregation and precipitation of free IgG, while IgG-QD maintained good colloidal stability due to its lower zeta potential than that of free IgG. Moreover, the optical properties, target recognition, and colloidal stability of the purified IgG-QD are maintained after salting out. This salting out strategy is suitable for the purification of the free IgG in IgG-QD solutions. This work facilitates the purification of QD-based bioprobes, and will contribute to the isolation of other impurities, such as the DNA or polypeptide in the bio-functionalization process of nanoparticles.

## Conflicts of interest

There are no conflicts to declare.

## Supplementary Material

NA-004-D1NA00569C-s001

## References

[cit1] Liu S. L., Wang Z. G., Xie H. Y., Liu A. A., Lamb D. C., Pang D. W. (2020). Chem. Rev..

[cit2] Zhang L. J., Xie H. Y., Xia L., Zhang Z. L., Pang D. W. (2019). Anal. Chem..

[cit3] Lv C., Zhang T. Y., Lin Y., Tang M., Zhai C. H., Xia H. F., Wang J., Zhang Z. L., Xie Z. X., Chen G., Pang D. W. (2019). Nano Lett..

[cit4] Wang W. T., Ji X., Kapur A., Zhang C., Mattoussi H. (2015). J. Am. Chem. Soc..

[cit5] Li J., Wang H., Lin L., Fang Q., Peng X. (2018). J. Am. Chem. Soc..

[cit6] Gianluca S., Simona S., Marianna M., Marco C., Stefano G., Antonella M., Stefano N., Marco F., Maria M., Debora B., Agnese M., Cristina N., Barbara R. (2018). Nanoscale.

[cit7] Wu J. K., Tian Z. Q., Zhang Z. L., Liu A. A., Tang B., Zhang L. J., Chen Z. L., Pang D. W. (2016). Talanta.

[cit8] Chen Z. L., Lin Y., Yu X. J., Zhu D. L., Guo S. W., Zhang J. J., Wang J. J., Wang B. S., Zhang Z. L., Pang D. W. (2017). ACS Appl. Mater. Interfaces.

[cit9] Mozhaev V. V., Heremans K., Frank J., Masson P., Balny C. (2015). Proteins: Struct. Funct. Genet..

[cit10] Bridgman P. W. (1914). Biol. Chem..

[cit11] Cioni P., Gabellieri E. (2011). BBA Proteins Proteom.

[cit12] Grover P. K., Ryall R. L. (2005). Chem. Rev..

[cit13] Laitinen J., Samarut J., Holtta E. (1994). Biotechniques.

[cit14] Mukerjee P., Chan C. C. (2002). Langmuir.

[cit15] Foster P. R., Dunnill P., Lilly M. D. (2010). Biotechnol. Bioeng..

[cit16] Brownlee Z., Lynn K. D., Thorpe P. E., Schroit A. J. (2014). J. Immunol. Methods.

[cit17] Ryu S., Lee B., Hong S., Jin S., Park S., Hong S. H., Lee H. (2013). Carbon.

[cit18] Foster P. R., Dunnill P., Lilly M. D. (1976). Biotechnol. Bioeng..

[cit19] Poh J., Gan S. (2014). Biosci. Rep..

[cit20] Grover P. K., Marshall V. R., Ryall R. L. (2003). Chem. Biol..

[cit21] Chen Z. L., Lin Y., Zhu C. N., Zhang Z. L., Pang D.
W. (2020). New J. Chem..

[cit22] Romano E. L., Mollison P. L. (2010). Vox Sang..

[cit23] Vo N. T., Ngo H. D., Vu D. L., Duong A. P., Lam Q. V. (2015). J. Nanomater..

[cit24] Pérez-Juan M., Flores M., Toldrá F. (2007). Food Res. Int..

[cit25] Mulligan A. M., Pinnaduwage D., Bane A. L., Bull S. B., O'Malley F. P., Andrulis I. L. (2010). Cancer.

[cit26] Sundqvist A., Asilaki E. V., Voytyuk O., Yu B., Dam H. V. (2020). Oncogene.

[cit27] Aiad H. A., Samaka R. M., Asaad N. Y., Kandil M. A., A Shehata M., Miligy I. M. (2014). Ecancermedicalscience.

[cit28] Selvi K., Badhe B. A., Papa D. (2014). Int. J. Surg. Pathol..

