# Supplementary material for: Purification of quantum dot-based bioprobes with a salting out strategy
Source: Nanoscale Adv. 2022 Jan 4;4(2):393–6. doi: 10.1039/d1na00569c (PMC9417310; doi:10.1039/d1na00569c)
Supplement: NA-004-D1NA00569C-s001 [file NA-004-D1NA00569C-s001.pdf]

Electronic Supplementary Material (ESI) for Nanoscale Advances.
This journal is © The Royal Society of Chemistry 2021

Purification of quantum dots-based bioprobes with salting out strategy

Zhi-Liang Chen [a], *Jia-quan Xu** [b]

[a] School of Pharmacy, Shaoyang University, Shaoyang, 422000, P. R China.

[b] Jiangxi Key Laboratory for Mass Spectrometry and Instrumentation, East China University of Technology, Nanchang, 330013, China

*E-mail: jiaquan_xu@foxmail.com. Tel: +86-739-5308282

Experimental Section

Materials

1-Octadecene (ODE, 90%), cadmium oxide (CdO, 99.99%), 1-octanethiol (98.5%), oleic acid (90%), trioctylphosphine (TOP), oleylamine (90%), 1-ethyl-3-(3-dimethylamino-propyl)-carbodiimide (EDC), polyacrylic acid (MW 1800), and selenium powder (Se, 99.99%) were purchased from Aldrich. Amine-PEG-carboxyl (NH_2-PEG-COOH, MW=2000) was purchased from Laysan Bio. Anti-rabbit second antibody and anti-mouse second antibody (MW=150 KDa) were purchased from Bioss Inc (Massachusetts, US). Other chemicals including sodium chloride (NaCl), hexane, N, N-dimethylformamide (DMF), chloroform, ethanol, and octylamine were purchased from China National Pharmaceutical Group Corporation. Ultrapure water (18 MΩ. cm^{-1}) was prepared by a Millipore Milli-Q system.

Synthesis of CdSe/CdS QDs

The CdSe/CdS QDs was prepared according to previous reports with minimal change.[1–3] Briefly, approximately 120 mg of CdO, 3 mL of oleic acid, 4 mL of oleylamine and 60 mL of ODE were added to a 250 mL three-necks flask. The resulting mixture was heated to 130 °C under vacuum for 20 min to remove oxygen and water. After purging 5 times with argon, the transparent solution were obtained and heated to 330 °C. Approximately 70 mg of Se powder dissolveed in 8 mL of TOP solution were injected into the three-necks flask swiftly. The resulting solution was rapidly cooled to 300 °C. Next, 1.0 mL of octanethiol dissolveed in 15 mL of ODE was injected into the three-necks flask at a flow rate of 1.2 mL/min. Finally, by removing heating source, the as-prepared CdSe/CdS QDs were cooled to room temperature. The CdSe/CdS were precipitated with the addition of ethanol and redispersed in hexane for water solubiliziation. CdSe/CdS QDs with green PL emission were prepared by controlling the amount of CdO and octanethiol.

Synthesis of OPA

Briefly, 1 g of polyacrylic acid and 1.5 g of EDC were dissolved in 50 mL of DMF. Approximately 700 μL of octylamine was injected into the flask at a flow rate of 1.0 mL/20 min and stirred for overnight under the argon atmosphere. Next, DMF was removed by rotary evaporation, and 20 mL of hydrochloric acid (1 mol/L) was added, and a sticky white precipitate was obtained. After washed three times with deionized water, the resulted OPA were dissolved in

ethanol and dried by rotary evaporation. Finally, the as-prepared OPA was stored in room temperature for the water-solubilization of CdSe/CdS QDs.

Preparation and OPA-QDs

Hydrophobic QDs (1 mg) and 5 mg of OPA were dispersed in 50 mL of chloroform. Subsequently, a OPA-QDs membrane layer was obtained by using a rotary evaporation to remove the chloroform. Adding 5 mL of borate saline buffer (pH 9, 50 mmol/L) to obtain OPA-QDs solution. Transmission electron microscope (TEM) images were obtained on a JEM2010FEF (UHR) microscope with an acceleration voltage of 200 kV. For the hydrophilic OPA-QDs samples, approximately 0.5 μL of sample (1×10^{-11} mol) mixed with 2 μL of phosphotungstic acid (2%) were dropped onto copper grids for TEM characterization. For hydrophobic samples, the CdSe/CdS QDs with appropriate concentration (1×10^{-11} mol/L) can be directly dropped onto the copper grids for the measurements. Agarose gel electrophoresis was performed on a DYY-6D gel electrophoresis apparatus (Beijing LiuYi instrument factory) with 1% (w/v) agarose gel, the gel picture was obtained by alphaimager HP system (Alpha Innotech). Dynamic light scattering measurements were carried out on a Malvern Nano instrument (ZS-ZEN 3600, Malvern Instruments, UK).

Salting-out of OPA-QDs solution

Approximately 1 mL of NaCl (2 mol/L) were added into 50 μL of OPA-QDs (3 μmol/L) solution, OPA-QDs solution change turbid aqueous phases. After 2 hours, the NaCl-treated OPA-QDs solution could be again recovered to transparent. The resulting samples were filtered by a centrifugal filter device with the MWCO of 50 kDa to remove NaCl, and the purified OPA-QDs was dispersed in borate saline buffer (pH 7.4, 20 mmol/L) and stored in room temperature for further characterization.

Preparation of OPA-QDs-PEG and QDs-IgG nanoprobes

The preparation of OPA-QDs-PEG and QDs-IgG nanoprobes was carried out according to our previous literature.[1] Briefly, 10 mg of EDC and 10 mg of NH_2-PEG-COOH (MW 2000) dispersed in borate saline buffer (pH 7.4, 20 mmol/L) were mixed with 2 mL of purified OPA-QDs (3×10^{-9} mol). After stirred at 160 rpm for 1.5 h, the resulting OPA-QDs-PEG were purified by gel filtration on NAP-10 column (GE Healthcare) to remove the excess of NH_2-PEG-COOH. QDs-IgG nanoprobes were prepared by conjugating IgG to the surface of OPA-QDs-PEG. Briefly,

1 mg of IgG and 100 μg of EDC dispersed in 1.5 mL borate saline buffer (pH 7.4, 20 mmol/L) containing OPA-QDs-PEG (1.0×10^{-9} mol). After stirred at 160 rpm for 2.0 h, the resulting QDs-IgG nanoprobes samples were stored in 5 °C. Based on the previous work,[4] we speculate that the conjugation efficiency is 50 %.

Salting-out of IgG-QD bioprobes

Typically, approximately 1 mL of NaCl (2 mol/L) were added into 200 μL of IgG-QD (3 μmol/L). After 8 hours, the resulting samples were filtered by a centrifugal filter device with the MWCO of 50 kDa to remove NaCl, the purified IgG-QD can be dispersed in borate saline buffer (pH 7.4, 20 mmol/L) again and stored in room 5 °C for future uses. The SEC included A Shodex KW-804 size exclusion column (Showa Denko scientific instruments (Shanghai) Co., Ltd.) was utilized to monitor the purification results. Borate saline buffer (pH 7.2, 20 mmol/L) containing NaCl (0.2 mol/L) was utilized as the mobile phase with a flow rate of 0.5 mL/min. Typical injection volume was 100 μL. An UV-Vis detector (SPD-20A, Shimadzu Corporation) at 210 nm was utilized to monitor the purification process.

QDs-Based Immunofluorescence Labeling

The QDs-Based immunofluorescence labelling procedure was carried out according to the instructions by Wuhan Jiayuan Quantum Dots Co., Ltd., Wuhan, China. The paraffin-embedded breast cancer tissues were provided as a gift from Hubei Cancer Hospital. Briefly, breast cancer tissues were dewaxed with xylene and dehydrated sequentially with 100%, 95%, 80%, and 70% alcohol. Two breast cancer tissues retrieve were performed in boiling EDTA (pH 9.0, 10 mM) solution for 3 min. Subsequently, one breast cancer tissues were incubated with rabbit anti-human *CK-8/18* monoclonal antibody, and the other breast cancer tissues were incubated with mouse anti-human *P-63* monoclonal antibody at 4 °C overnight. Washed three times with TBS-T (0.1 M Tris-base, 0.5% Tween, 0.8% NaCl, pH 7.6) to remove the excess primary antibodies. Then the as-prepared two kinds of QDs-IgG nanoprobes were added to incubate respectively with *CK8/18* and *P-63* on breast cancer tissues at 37 °C for 1 h, and washed three times with TBS-T to remove the excess QDs-IgG nanoprobes. The fluorescence signal of QDs was collected using the Caliper multispectral microscopy imaging system (Caliper Life Sciences, Hopkinton, MA, USA).

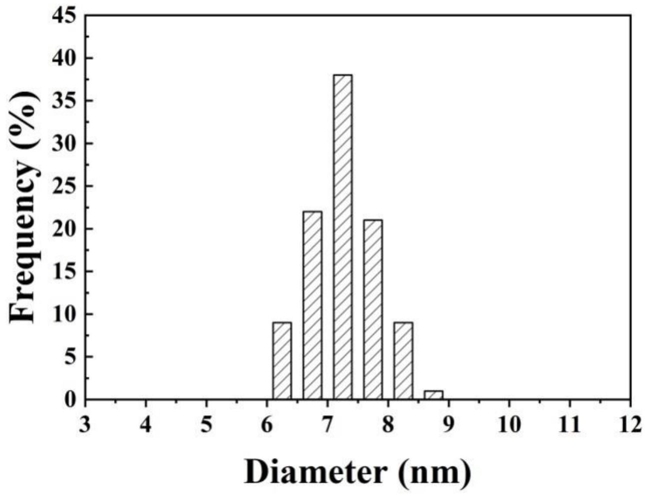

Fig. S1 Size distribution histograms of the as-prepared CdSe QDs, the corresponding size is 7.3 ± 1.2 nm, the sizes are from measuring at least 150 individual QDs in TEM images

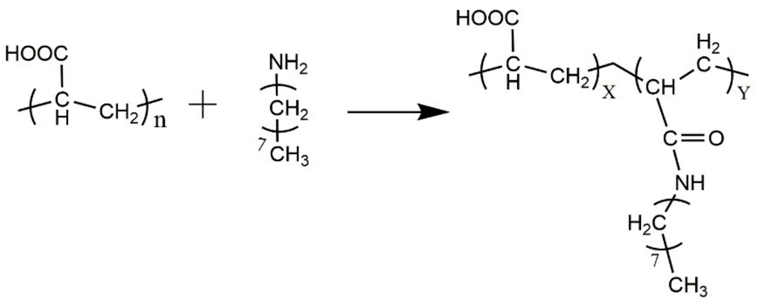

Fig. S2 Schematic diagrams for the synthesis of Octylamine-grafted poly-(acrylic acid) (OPA)

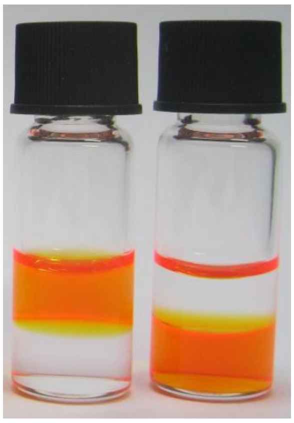

Fig. S3 picture of QDs before and after coated with OPA (left: QDs dispersed in hexane, right: OPA-QDs dispersed in water)

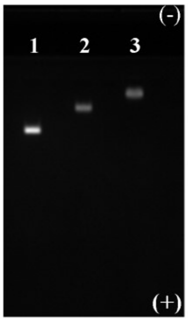

Fig. S4 Agarose gel electrophoresis image of OPA-QDs (1), OPA-QDs-PEG (2) and IgG-QD bioprobes (3)

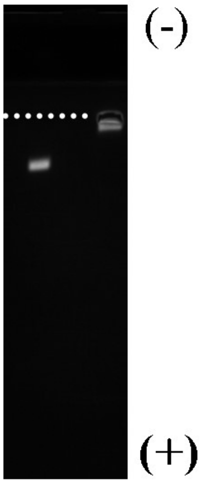

Fig. S5 Agarose gel electrophoresis image of IgG-QD before (left, IgG-QD) and after (right, NaCl-treated IgG-QD) adding NaCl, the arrow represents the position of loading well.

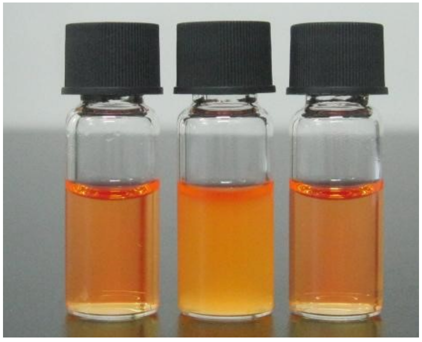

Fig. S6 picture of IgG-QD before and after treated NaCl (left: IgG-QD dispersed in BR buffer; middle: IgG-QD treated NaCl; right: NaCl removed)

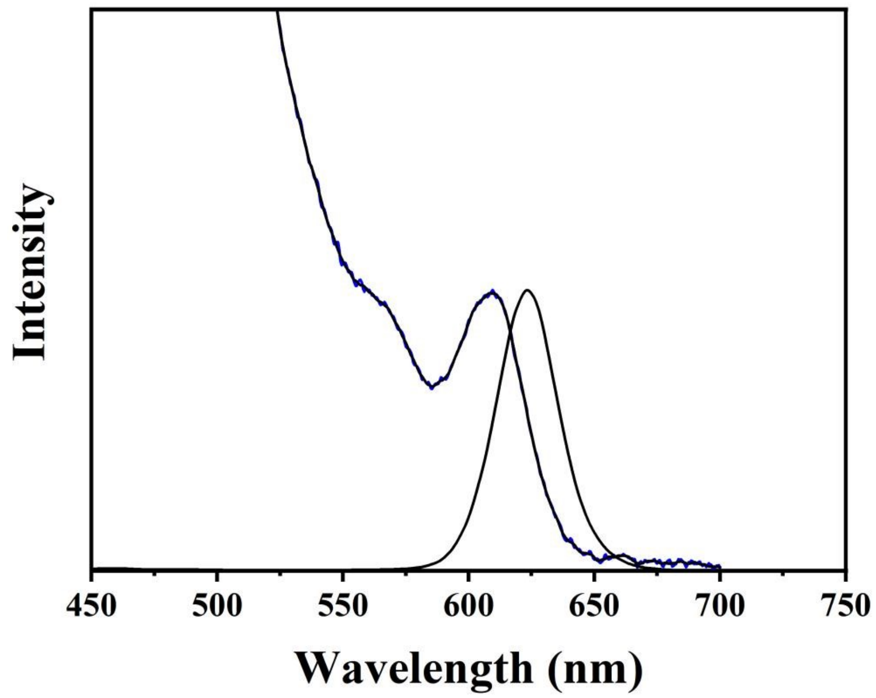

Fig. S7 the absorption and PL spectra of IgG-QD before and after adding NaCl.

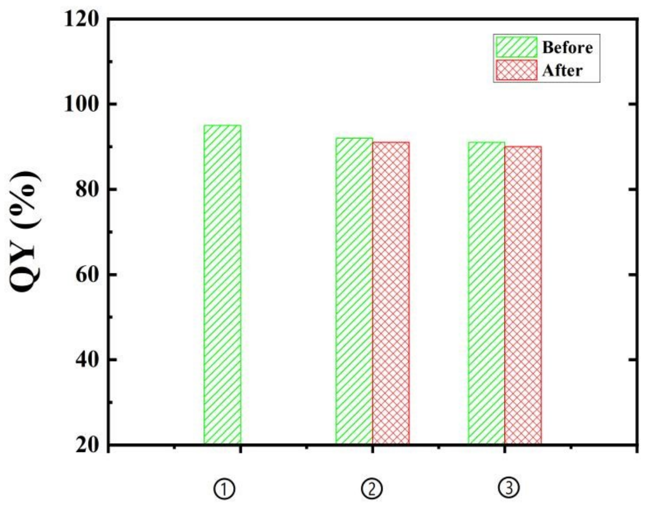

Fig. S8 Quantum yield of QDs samples before and after adding NaCl. ①: hydrophobic CdSe/CdS QDs dispersed in hexane, ②:OPA-QDs before (green) and after (red) treated with NaCl, ③: IgG-QD bioprobes before (green) and after (red) treated with NaCl.

References

1 Z. L. Chen, Y. Lin, C. N. Zhu, Z. L. Zhang, D. W. Pang, *New J. Chem*. 2020, **44**, 15341−15344.
2 Z. L. Chen, Y. Lin, X. J. Yu, D. L. Zhu, S. W. Guo, J. J. Zhang, J. J. Wang, B. S. Wang, Z. L. Zhang, D. W. Pang. *ACS Appl. Mater. Interfaces*, 2017, **9**, 39901−39906.
3 W. T. Wang, X. Ji, A. Kapur, C. Zhang, H. Mattoussi. *J. Am. Chem. Soc.* 2015, **137**, 14158−14172.
4 Y. Xing, Q. Chaudry, C. Shen, K.Y. Kong, H. E. Zhau, L. W. Chung, J. A. Petros, R.M. O'Regan, M. V. Yezhelyev, J. W. Simons, M. D. Wang. S. Nie. *Nat. Protoc*. 2007, **2**, 1152−1165.
